# Highly Sensitive Temperature Sensors Based on Fiber-Optic PWM and Capacitance Variation Using Thermochromic Sensing Membrane

**DOI:** 10.3390/s16071064

**Published:** 2016-07-09

**Authors:** Md. Rajibur Rahaman Khan, Shin-Won Kang

**Affiliations:** School of Electronics Engineering, Kyungpook National University, 80 Daehakro, Bukgu, Daegu 41566, Korea; rajibur@ee.knu.ac.kr

**Keywords:** temperature sensor, fiber-optic pulse width modulation, Rhodamine-B, interdigitated capacitor, sensing membrane, sensing element, dielectric constant

## Abstract

In this paper, we propose a temperature/thermal sensor that contains a Rhodamine-B sensing membrane. We applied two different sensing methods, namely, fiber-optic pulse width modulation (PWM) and an interdigitated capacitor (IDC)-based temperature sensor to measure the temperature from 5 °C to 100 °C. To the best of our knowledge, the fiber-optic PWM-based temperature sensor is reported for the first time in this study. The proposed fiber-optic PWM temperature sensor has good sensing ability; its sensitivity is ~3.733 mV/°C. The designed temperature-sensing system offers stable sensing responses over a wide dynamic range, good reproducibility properties with a relative standard deviation (RSD) of ~0.021, and the capacity for a linear sensing response with a correlation coefficient of R^2^ ≈ 0.992 over a wide sensing range. In our study, we also developed an IDC temperature sensor that is based on the capacitance variation principle as the IDC sensing element is heated. We compared the performance of the proposed temperature-sensing systems with different fiber-optic temperature sensors (which are based on the fiber-optic wavelength shift method, the long grating fiber-optic Sagnac loop, and probe type fiber-optics) in terms of sensitivity, dynamic range, and linearity. We observed that the proposed sensing systems have better sensing performance than the above-mentioned sensing system.

## 1. Introduction

Temperature sensors are useful (even essential) devices in many areas of our lives. They are primarily used to measure and monitor the temperature driven by several environmental conditions. Temperature sensors have applications in various fields such as medicine/biomedicine, the food and beverage industry [1], agriculture and horticulture [2], industrial processing, and research and development [3]. In the field of medicine [4,5], temperature sensors are used in real time structural health monitoring (SHM), kidney dialysis machines, organ transplant systems, and medical incubators. In the agricultural sector, they are used for monitoring the temperature of plants, soil, and water [6]. In the food and beverage industry temperature sensors are used in fermentation, brewing, meat processing, and the fabrication of storage tanks. Temperature sensors are also used in the petrochemical industry, the automotive industry, metal industries, geothermal wells, the consumer electronics industry, petroleum industries, and in harsh environmental applications [7].

Over the last several decades, many sensors have been designed to detect temperature, such as capacitive [8,9], surface acoustic wave [10,11], carbon nanotube [12,13], Schottky diode [14], BJT-MOSFET pair [15], CMOS [16], surface Plasmon resonance (SPR) [17], and fiber-optic [18] sensors.

Recently, fiber-optic temperature sensors have been shown to be excellent candidates because they offer advantages such as low weight, small dimensions, geometrical versatility, remote-sensing capabilities, freedom from electromagnetic interference, and complete electrical isolation. Therefore, they are highly safe and can sense multiple parameters using a single optical fiber with no cross talk. To date, many fiber-optic temperature sensors have been developed, such as the side-polished fiber-optic temperature sensor [19], U-shaped temperature sensor [20], D-type fiber-optic temperature sensor [21], fiber-optic Fabry-Perot temperature sensor [22], and fiber-optic Bragg-grating temperature sensor [23].

However, interdigitated electrode (IDE)-based sensors are in high demand at present. These sensors are used in a broad range of applications; for example, gas sensors [24], biosensors [25,26], pH sensors [27], chemical sensors [28], taste sensor [29,30], glucose sensors [31], humidity sensors [32], pressure sensors [33], and temperature sensors [34].

Mariam-deme Dankoco et al. proposed a flexible, inkjet-printed IDE-based temperature sensor to measure the temperature of the human body [35]. The sensor works on the resistive variation principle under a varied temperature. The construction and principle of operation of the sensor are very simple; it works at a bias voltage of 1 V and has a small size. However, the proposed sensor has a low dynamic range of 20–60 °C and less stable performance. 

A fiber-optic MEMS temperature sensor was developed by Yixian Ge et al. in 2016 [36]. The operation principle of the sensor is based on optical Fabry-Perot interference and the bimetallic diaphragm effect. Although, their proposed temperature sensor has some advantages, it also has several disadvantages such as a complex fabrication procedure and a low dynamic range of 20–70 °C. Ying Wang et al. proposed an ultrahigh sensitivity photonic crystal fiber temperature sensor in 2011 [37]. In their study, they create several air holes in the photonic crystal fiber; then, they fill one of the air holes with a standard refractive index liquid to create the sensor. The main feature of this sensor is its ultrahigh sensitivity, which is ~54.3 nm/°C. However, the proposed temperature sensor also has disadvantages such as a complex fabrication process and a very short dynamic range of approximately 34–35.4 °C. 

Evanescent field coupling between a single-mode side-polished optical fiber and a polymer waveguide overlay on the side-polished optical fiber was proposed by Alberto Álvarez-Herrero et al. [38]. In this work, the temperature was measured by observing the resonance wavelength shift. Although the temperature sensor offers good sensitivity and linearity, it also has some disadvantages for example low dynamic range (26 °C to 40 °C) and difficulty of obtaining the resonance wavelength. Ruan proposed a Sagnac-loop-based fiber-optic sensor to measure temperature from 20 °C to 50 °C in 2015 [39]. In his work, he fabricated the sensor head by combining a single-mode/multimode/polarization-maintaining fiber with a tilted long-period fiber grating. The proposed temperature has good sensing ability and linearity; however, its temperature-sensing range is low and it has a complex fabrication process.

In our experiment, we proposed a side-polished fiber-optic temperature sensor that is based on the principle of fiber-optic pulse width modulation (PWM) [40,41,42] technique. Using this principle, the received sensing signal pulse width changes as the side-polished fiber-optic temperature-sensing element is heated. A thermochromic dye, namely, Rhodamine-B, was mixed with a polymer and an *N*,*N*-dimethylacetamide (DMAC) solution to create a dielectric/thermally sensitive material. Then, this material was deposited on the side-polished fiber-optic device using a spin coater to create a fiber-optic sensing element. In our study, we also prepared an interdigitated capacitor (IDC) temperature sensor that is based on the capacitance variation principle as the IDC sensing element is heated. When the sensing elements (fiber-optic and IDC) are heated, the refractive index and dielectric constant of the sensing membrane change. As a result, the received sensing signal’s pulse width for the case of a fiber-optic sensing element changes. In the case of the IDC temperature-sensing element, the capacitance of the IDC sensing element changes. The proposed temperature sensors have many advantages including low-cost, compactness, ease of fabrication, high sensitivity, highly stable response performance, wide dynamic range, fast response and recovery times, and ease of manufacture (the designed circuitry can be prepared from easily obtainable, low-cost electronic components). Finally, we compare the performance of our proposed fiber-optic PWM and IDC temperature sensor with that of other sensors in terms of parameters (sensitivity, dynamic range, and linearity); we found that our proposed sensors offer better sensing performance than others. 

## 2. Theory and Working Principle

In our study, we applied two different sensing principles (i.e., fiber-optic PWM and capacitance variation) to prepare different types of temperature sensor to measure the temperature from 5–100 °C.

### 2.1. Theory and Working Principle of the Fiber-Optic PWM Temperature Sensing System 

**Figure 1:**
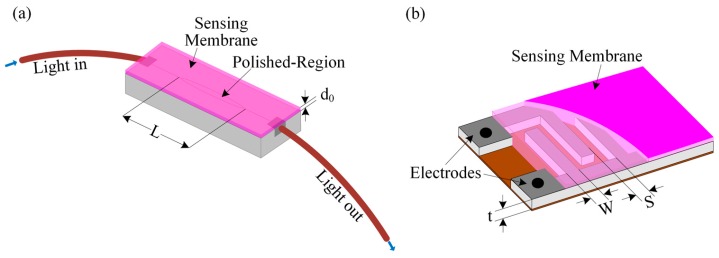
Schematic diagram of the temperature-sensing elements: (**a**) Side-polished optical fiber with a sensing membrane; and (**b**) IDC sensing element.

A sensing membrane containing Rhodamine B was deposited on a side-polished optical fiber to form an optical waveguide; the schematic diagram is shown in Figure 1a. When light travels through the side-polished fiber-optic device, a fraction of the radiation spreads a small distance to create an evanescent field, as defined by [43]:

(1) $$E\left(z\right)=E_{0}\text{exp}\left(-\frac{z}{d_{P}}\right)$$

where $E_{0}$ is the electric field amplitude of light at the interface of core–cladding and z is the distance of electric field in the cladding from the interface. $d_{p}$ is the penetration depth, and the sensitivity of the fiber-optic sensor depends on the penetration depth. The energy of the evanescent field may change because of changes in the refractive index of the overlay waveguide or changes in the absorption or scattering of light into the overlay waveguide. Mathematically, penetration depth can be represented by [44]:

(2) $$d_{p}=\frac{\lambda }{2\pi {\text{n}}_{1}}{\left\{\sin^{2}\theta -{\left(\frac{n_{2}}{n_{1}}\right)}^{2}\right\}}^{-0.5}$$

where λ and θ are the wavelength of the transmitted light and the angle of incidence to the normal at the interface, respectively. $n_{1}$ is the refractive index of the fiber cladding. $n_{2}$ is the refractive index of the material in contact with the top surface of the overlay. 

An electrical PWM system generally has two inputs—a pulse input, which is used to input the pulse into the system; and a control input, which is used to change the pulse width of the input signal without changing the time period of the input signal—and one output, which is used to obtain the desire pulse width. In our proposed fiber-optic PWM system, we send a light pulse through a fiber-optic-based polymer waveguide that contains a thermochromic compound. If the overlay waveguide containing a thermochromic compound is heated, then its optical properties (for example, refractive index) change. As a result, the peak value of the pulse and the fall time change because of the light absorption in the waveguide, which, in turn, changes the pulse width of the received sensing signal. The output received pulse width relies on light absorption within the overlay polymer waveguide, which can be taken into account as a pulse control input. This is because the absorption of the light pulse occurs due to the change in the refractive index of the overlay waveguide, which corresponds to the change in temperature. The received light pulse width $T_{H}$ can be written as [40,41]:

(3) $$T_{H}=\frac{T}{n{\left(\frac{\gamma \text{L}}{\alpha }\right)}^{0.5}}$$

where T and L are the time period of the light wave and the length of the polished cladding, respectively. γ is the evanescent wave absorption coefficient, α is the phenomenological ion-specific parameter, and n is the refractive index of the overlay waveguide. Therefore, by measuring the pulse width of the received signal, the amount of heat can be determined. The pulse width is proportional to the heat absorbed by the thermochromic-compound-based polymer waveguide, which corresponds to the absorption of the evanescent field into the wave guide.

### 2.2. Theory and Working Principle of the IDC Temperature Sensing System

Figure 1b shows a schematic diagram of an IDC consisting of finger/comb-shaped electrodes covered with the thermochromic-compound-based dielectric or sensing membrane. An AC voltage source is applied to the two terminals of the IDC to generate an electric field; these electric lines of flux penetrate the dielectric or the sensing membrane. The total capacitance of the IDC can be expressed mathematically as follows [30]:

(4) $$C=l\left(N-1\right)\left\{\frac{\varepsilon_{0}\varepsilon_{r}}{2}\frac{K{\left({1-k}^{2}\right)}^{0.5}}{\text{K}(\text{k})}+2\varepsilon_{0}\varepsilon_{r}\left(\frac{t}{S}\right)\right\}$$

where N, l, S, and t are the number of fingers, the length of the IDE, the spacing between two adjacent fingers of the IDE, and the thickness of the electrode, respectively. $\varepsilon_{0}$ is the absolute dielectric constant and $\varepsilon_{r}$ is the relative dielectric constant of the sensing membrane or medium. K(k) is the elliptic integral of the first kind of modulus k, which can be expressed as:

(5) $$k=\cos\left\{\frac{\pi W}{2(W+S)}\right\}$$

where W is the width of the electrode.

Therefore,

(6) $$\text{K}(\text{k})=\int_{0}^{1}\frac{\text{K}(\text{k})}{{\left\{\left(1-t^{2}\right)\left(1-k^{2}t^{2}\right)\right\}}^{0.5}}\text{dt}$$

Because the proposed sensor is based on the principle of the capacitor, the voltage across the IDC sensing element is defined as:

(7) $$v_{C}=\frac{i_{C}}{2\pi fC}$$

where $i_{C},f,\mathrm{and}C$ are the current flowing through the IDC sensing element, the frequency of the applied signal, and the capacitance of the IDC sensing element, respectively. When the IDC-sensing element absorbs heat from any source, its dielectric constant changes, which corresponds to changing both the capacitance and the voltage across the IDC sensing element; this fact can be represented by the following equation:

(8) $$\Delta v_{C}=\frac{i_{C}}{2\pi f\Delta C}$$

## 3. Experimental Details

### 3.1. Fabrication of the Side-Polished Optical Fiber Device

**Figure 2:**
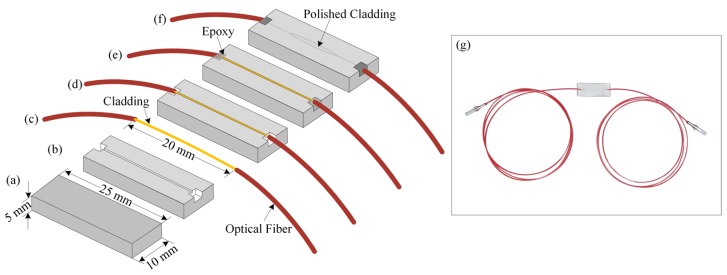
Step-by-step fabrication procedure of the side-polished fiber-optic device: (**a**) Quartz block; (**b**) Creating the V-groove on the quartz block; (**c**) Removing the jacket of the optical fiber; (**d**) Bending the removed-jacketed-portion of the optical fiber and placing it into the V-groove of the quartz block; (**e**) Using epoxy; (**f**) Polishing the fiber-optic quartz block; and (**g**) A photograph of the fabricated side-polished fiber-optic device.

In our experiment, we prepared a side-polished fiber-optic device. We selected a quartz block of size 25 × 10 × 5 mm and made a V-groove of 160 µm in width on the quartz block using a mechanical slicer. Then, we removed the jacket, which had a length of ~20 cm, of this single-mode optical fiber. The radii of the core and cladding of the used single-mode optical fiber were 3 µm and 125 µm, respectively. Then, we bent the removed jacket with a radius of ~60 cm and placed it in the V-grooved quartz block. Then, we applied epoxy and dried the epoxy to strongly attach the fiber to the quartz. We used l000-μm and 8000-μm polishing powders on polishing pads to polish the surface of the cladding that was attached to the quartz block to create a side-polished fiber-optic device. The step-by-step fabrication process of the side-polished fiber-optic device is shown in Figure 2a–f. The length of the fiber was 1 m. A photograph of a side-polished fiber-optic device is shown in Figure 2g.

### 3.2. Fabrication of the IDE 

**Figure 3:**
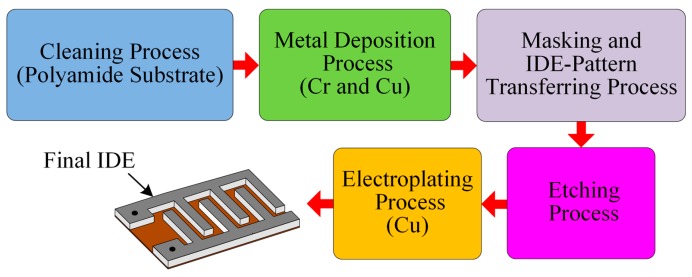
Fabrication stages of the IDE.

We prepared IDEs to form an IDC-based temperature-sensing element. To make an IDC temperature-sensing element, we first had to fabricate an IDE. Then, we deposited Rhodamine-B including sensing membrane into the IDE to make IDC. We prepared an IDE with a thickness of approximately 22 µm on a 4 × 2 cm polyimide (PI) substrate with 40 pairs of fingers. We applied the vacuum evaporation method to make a thin film of Cr and Cu on the PI substrate, respectively. The thicknesses of the Cr and Cu films were approximately 10 nm and 15 nm, respectively. The IDE’s photomask pattern was transferred onto the thin metal film. Then, a chemical etchant was used to etch the unmasked pattern to obtain thin IDE fingers on the PI substrate. We applied a Cu electroplating method to increase the thickness of the Cu electrodes. Finally, we cut the residual PI substrate. Figure 3 shows the various stages used to fabricate the IDE. To measure the IDE thickness, the width of the fingers, and the distance between fingers, we used a scanning electron microscope (SEM) (S-4800, Hitachi, Ibaraki, Japan); the measured values were approximately 22 μm, 100 μm, and 100 μm, respectively. 

### 3.3. Fabrication of the Sensing Membrane, Fiber-Optic Sensing Element, and Interdigitated Capacitor

**Figure 4:**
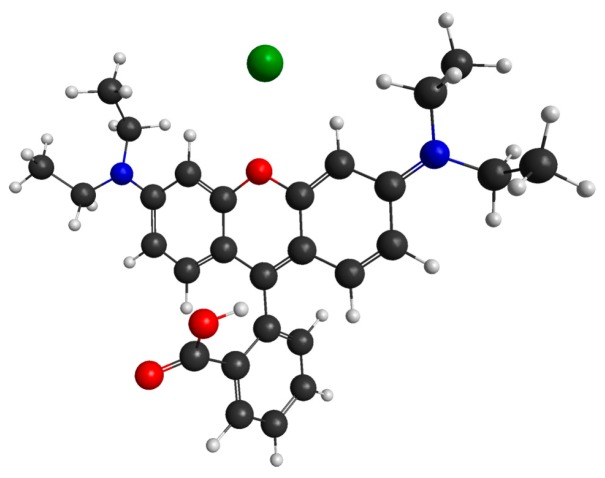
Molecular structure of the Rhodamine-B thermochromic dye.

**Figure 5:**
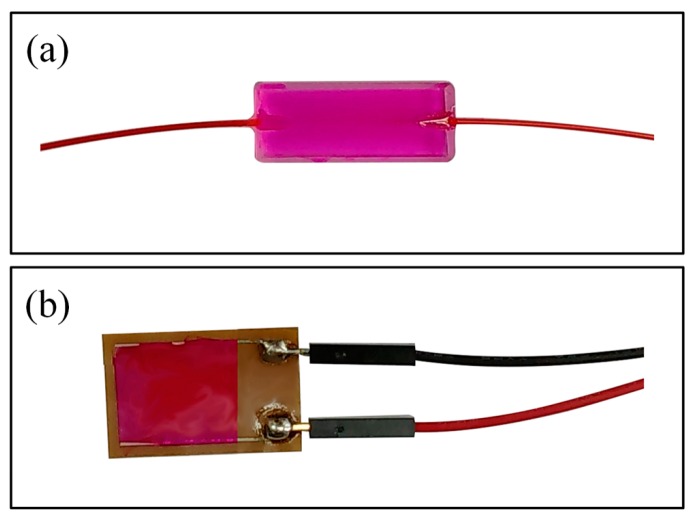
Fabricated temperature-sensing elements after deposition on the sensing membrane: (**a**) Fiber-optic and (**b**) IDE.

The capability of a chemical compound to change color owing to a change in temperature is called thermochromism; a chemical compound that exhibits this property is called a thermochromic dye [45]. To create the temperature-sensitive sensing membrane, we used Rhodamine B [46,47] thermochromic dye, a polymer, namely, polyvinyl chloride (PVC), and a DMAC solution. The molecular structure of the thermochromic dye (Rhodamine B) is shown in Figure 4. We obtained chemicals from the Sigma-Aldrich Chemical Corporation (Seoul, Korea) and used them without any further purification. First, 0.020 g of Rhodamine B was dissolved in the DMAC solution. DMAC is an aprotic solution; therefore, it does not react with the dye molecule. Then, we added 0.035 g of PVC powder to the Rhodamine B solution and sonicated it for 10 min to obtain a temperature-sensitive dielectric solution. Before depositing the sensing solution, we cleaned the side-polished fiber-optic device and IDE with ethanol, methanol, and deionized (DI) water, respectively. Then, we dried both the side-polished fiber-optic and the IDE in N_2_ gas. We used a syringe to take 0.25 mL of sensing solution to deposit the solution on the side-polished fiber-optic and the IDE. Then, we used a spin coater to deposit the solution smoothly on the side-polished fiber-optic and IDE and dried the devices at room temperature to obtain fiber-optic and IDC temperature-sensing elements. The fabricated fiber-optic and IDE temperature-sensing elements, after deposition on the sensing membrane, are shown in Figure 5a,b, respectively. 

In our experiment, we used the PVC polymer to immobilize the Rhodamine B on the side-polished optical fiber. Since the polished surface of the optical fiber is very low, it is difficult to deposit the Rhodamine B containing PVC polymer membrane on the side-polished optical fiber. Therefore, we attached a quartz block to the side-polished portion of the optical fiber, which increased the surface area to allow the temperature sensitive sensing membrane to be deposited properly. We used the PVC polymer, whose melting point is about 240 °C. Therefore, there is no significant effect of temperature on the polymer as well as the quartz block for the proposed sensing systems.

### 3.4. Detection Mechanism of the Proposed Temperature Sensing System

**Figure 6:**
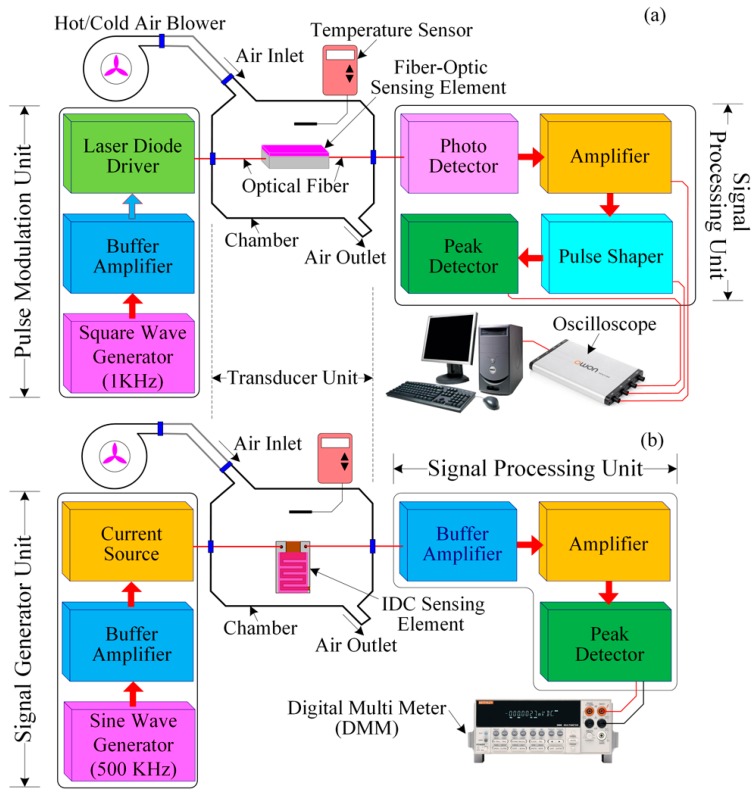
Schematic diagram of the experimental setup of the proposed temperature sensing system: (**a**) Fiber-optic PWM and (**b**) IDC.

Figure 6a shows the experimental setup for the characterization of the fiber-optic PWM temperature-sensing system. The proposed fiber-optic PWM temperature-sensing system consists of three units: a pulse modulation unit, a transducer/temperature-sensing unit, and a signal processing unit. We have designed the pulse modulation unit and the signal-processing unit using low-cost and easily obtainable electronic components from local electronics suppliers. The pulse modulation unit consists of a square wave generator, which generates a 1-kHz square wave with a 50% duty cycle, a buffer amplifier, a laser diode (LD) driver, and an LD, which emits at an 850-nm peak wavelength. The buffer amplifier has low input impedance and high output impedance, which is used to reduce loading effects. We used an NE555 timer and associated passive electronic components to design the square wave generator; it generates a square wave at a frequency of ~2 kHz without a 50% duty cycle. Then, its output was passed through a T-flip-flop (CD4027, JK flip-flop employed in toggle mode) to obtain a perfect 1-kHz signal with 50% duty cycle. The output of the flip-flop is connected to the input of the LD driver circuit via the buffer amplifier to obtain the 1-kHz light pulse. This light pulse is passed through the side-polished fiber-optic temperature-sensing element and its output is connected to the signal processing unit. 

The signal processing unit can be divided into four parts: a light/photo detector (PD), an amplifier, a pulse shaping circuit, and a peak detector. The optical signal from the side-polished fiber-optic temperature-sensing element was detected by the photodiode. The output of the PD is connected to an operational amplifier for amplification. The op-amp is connected in the current follower configuration. Then, the output of the amplified signal is fed to the input of the pulse shaping circuit. Then, the output signal of the pulse shaping circuit is fed to the input of the peak detector. The peak detector is used to obtain the peak value of the signal. The fiber-optic sensing element contains thermochromic dye including the sensing membrane; therefore, when the sensing membrane is heated, the refractive index of its overlay waveguide changes (as a result the pulse width of the received sensing signal changing). Therefore, the relative pulse width (ΔT_H_) is the difference between the reference pulse width and the sensing pulse width for a given temperature. The relative pulse width increases as the temperature increases. An oscilloscope (OWON, VDS3104, Guangzhou, China) was used to observe both the pulse width variation due to the change in temperature and the output voltage of the peak detector. 

Figure 6b shows a schematic diagram of the experimental setup of the proposed IDC temperature-sensing system. The IDC sensing element contains Rhodamine B dielectric material; therefore, when the IDC sensing element is heated, its dielectric constant and the capacitance of the IDC change (as a result the output voltage of the peak detector changing). The output voltage is measured using a digital multimeter (DMM) (Keithley, 2002, Cleveland, OH, USA). 

In our study, we developed the hot/cold air blower which could provide accurate air temperature from 3–100 °C. We took temperature measurements using the proposed systems from 5–100 °C. To observe the temperature below 5 °C we put an ice pack on the fiber-optic/IDC temperature sensing element of the proposed temperature sensing system and measured the ice pack temperature using a commercial thermocouple thermometer (ET-959, Shenzhen, China). The observed temperature was 0 °C. Then we observed the sensing ability of the proposed fiber-optic PWM and IDC temperature sensing system at 0 °C. We found that the proposed sensing systems also gave a response at 0 °C. The relative pulse width and the capacitance of the proposed fiber-optic PWM and IDC temperature sensing system at 0 °C were about 0.06 µs and 1.73 nF, respectively. From this experiment, we found that the proposed temperature sensing systems is also able to measure the temperature below 5 °C. 

There is still a controversy about the innocuousness of Rhodamine B. Therefore, if we want to use the proposed sensing system for medical applications, the whole fiber-optic or IDC temperature sensing element must be covered with a thin layer of medical grade plastic that has a high melting point. This keeps the Rhodamine B containing the sensing membrane from coming into direct contact with the human body. We believe that this situation will not affect the temperature sensing performance of the proposed sensing systems. Because when the fiber-optic/IDC sensing elements, which will be covered with the medical grade plastic, receives heat from any source, the Rhodamine B containing temperature sensitive membrane also receives heat. As a result, the refractive index of the Rhodamine B containing the sensing membrane will change, which will change the pulse width, as well as the capacitance of the proposed fiber-optic PWM and IDC sensing system, respectively. The proposed temperature sensors are practical and cost effective single-point temperature sensors.

## 4. Results and Discussions

**Figure 7:**
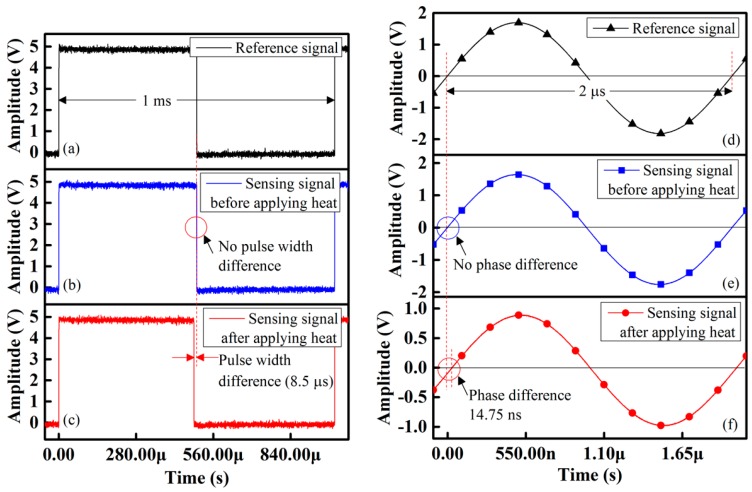
Waveform response of the proposed temperature sensors: (**a**) Reference signal of fiber-optic PWM sensing system; (**b**) Fiber-optic PWM sensing system before applying heat; (**c**) Fiber-optic PWM sensing system after recording heat response after applying heat; (**d**) Reference signal of IDC sensing system; (**e**) IDC sensing system before applying heat; and (**f**) IDC sensing system after applying heat.

Figure 7a,b shows the reference and sensing waveforms at 3 °C for the case of a fiber-optic PWM temperature-sensing system; we consider this value to be a reference temperature. Figure 7b shows that there was no change of pulse width of the sensing signal with respect to the reference signal. However, once the side-polished fiber-optic sensing element is heated, the pulse width of the sensing signal changes, as shown in Figure 7c. The relative pulse width difference at 50 °C was ~8.5 µs; the result is shown in Figure 7c. This result indicates that the sensing response of our designed signal processing unit is good and that it has the ability to detect small differences in the pulse width. 

**Figure 8:**
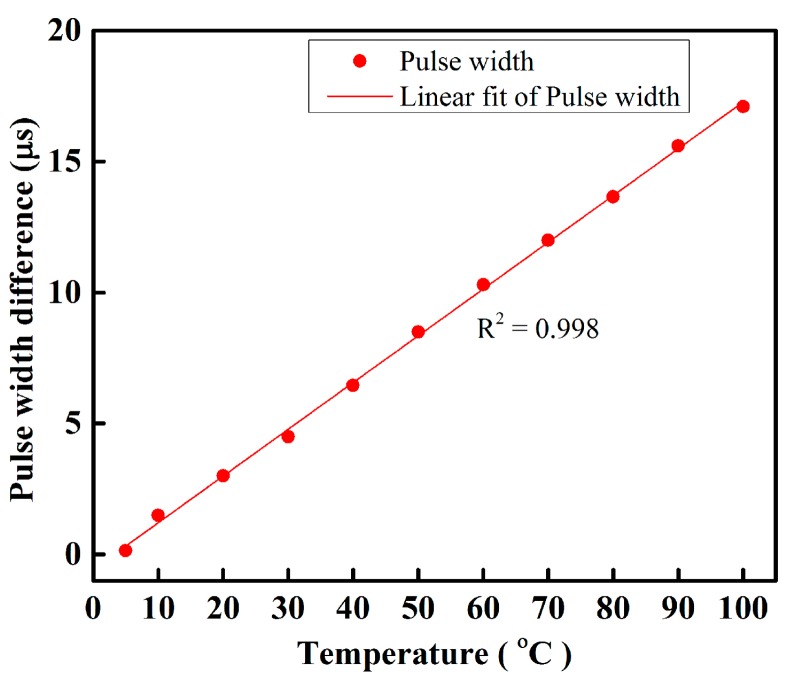
Behavior of the proposed fiber-optic PWM temperature sensing system under different temperatures.

We also prepared an IDC temperature sensor to measure the temperature from 5–100 °C. The capacitance of the IDC sensing element, the capacitive impedance, and the phase shift of the received signal of the proposed IDCs are functions of the temperature variation. The waveform response for phase shifting is shown in Figure 7e; we found that at 3 °C, there is no phase difference; however, when the temperature increases, a phase shift occurs between the received sensing and reference signals. The phase shift difference between the received sensing and reference signals at 50 °C was ~14.75 ns and was measured using an oscilloscope; the results are shown in Figure 7f. The temperature sensing ability of the proposed fiber-optic PWM sensing system under different temperature is shown in Figure 8. From this figure, we found that the relative pulse width linearly increases as the temperature increases and its correlation coefficient R^2^ was about 0.998 with a high temperature sensing ability. The sensitivity of the proposed fiber-optic PWM sensing system was ~178 ns/°C.

**Figure 9:**
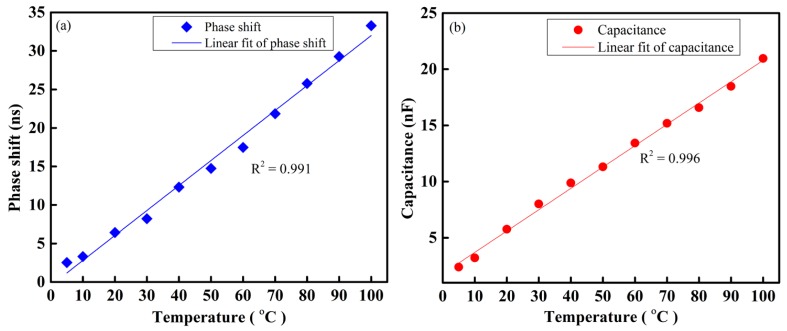
Behavior of the proposed IDC thermal/temperature sensing system under different temperatures: (**a**) Phase shift and (**b**) Capacitance.

The phase shift difference between the sensing signal and the reference signal at different temperatures from 5–100 °C is recorded, as shown in Figure 9a. We find that the phase shift between the two signals increases linearly with temperature. The variation of capacitance with respect to the different temperature is shown in Figure 9b.

**Figure 10:**
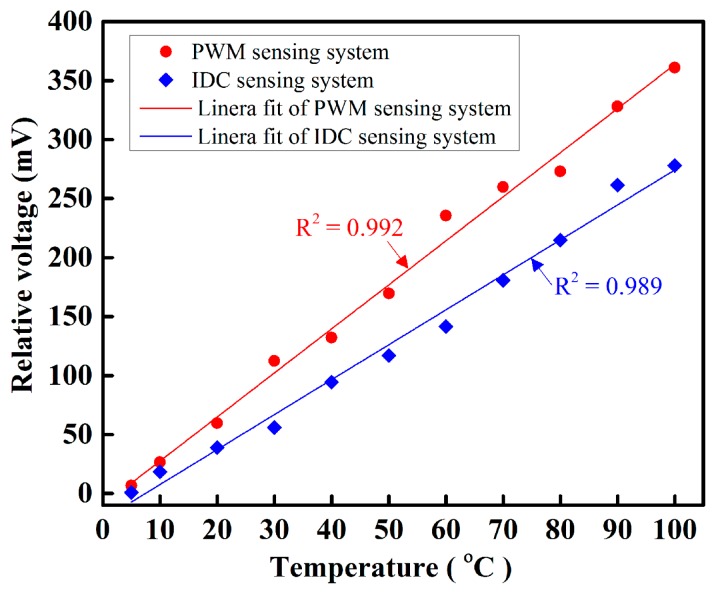
Temperature response of the proposed sensing systems.

Figure 10 shows the sensing performance of the proposed temperature-sensing systems. This figure shows that as the temperature increases the relative voltage linearly increases. In Figure 10, we used linear curve fitting to determine the slope, i.e., the sensitivity of the proposed fiber-optic PWM and IDC temperature-sensing systems. The sensitivity of the proposed fiber-optic PWM and IDC temperature-sensing systems was 3.733 mV/°C and 2.96 mV/°C, respectively. From the above described results, we determined that the fiber-optic PWM sensing system offers greater sensing ability than the IDC temperature-sensing system. 

**Figure 11:**
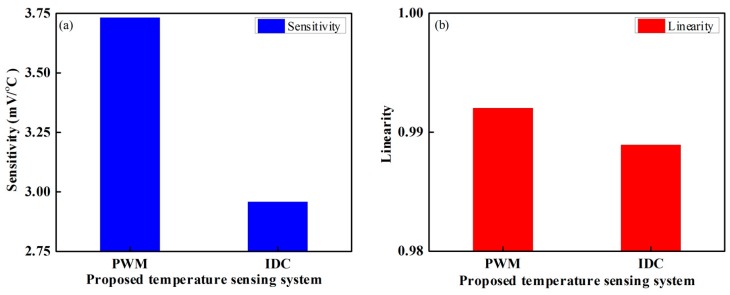
Graphical representation of the proposed fiber-optic PWM and IDC temperature sensing system: (**a**) Sensitivity and (**b**) Linearity.

In our experiment, we tried to measure the linearity of the proposed fiber-optic PWM and IDC temperature sensors over the dynamic range from 5–100 °C; the device had good linearity. The correlation coefficient (R^2^) values for the proposed fiber-optic PWM and IDC temperature sensors were approximately 0.992 and 0.989, respectively. Figure 11 shows the graphical representation for sensitivity and linearity of the proposed fiber-optic PWM and IDC temperature-sensing system.

We created three samples of fiber-optic PWM temperature-sensing elements. Then, we observed the sensing ability of these fiber-optic sensing elements at 50 °C to determine the reproducibility performance. We observed that the three devices had almost the same sensing performance. Therefore, the sensing elements have excellent reproducibility; their relative standard deviations (RSDs) were approximately 0.021.

To observe the repeatability of the proposed fiber-optic PWM temperature sensing system, our experiment was repeated three times at different temperatures. The relative pulse width for the measurements was recorded. We found that there was no significant variation of the pulse width at the specified temperature using the proposed fiber-optic PWM temperature sensing system. For example, the relative pulse width at 50 °C for three observations were 8.50 µs, 8.506 µs, and 8.492 µs. Therefore, we can say that the proposed temperature sensing system has an excellent repeatability response. According to our experimental observations, the response and recovery times of the proposed temperature sensors were 5 s and 6 s, respectively.

We compared the performance of the proposed temperature-sensing systems with different temperature sensors. The sensitivities of the proposed fiber-optic PWM and IDC temperature sensors were approximately 3.733 mV/°C and 2.96 mV/°C, respectively, whereas the sensitivities of the fiber-optic wavelength shift temperature sensor [38], fiber-optic Sagnac loop sensor [39], and probe type fiber-optic temperature sensor [48] were approximately −0.97 nm/°C, −1.5 nm/°C, and 0.0044 mV/°C, respectively. Moreover, the dynamic ranges of the proposed sensors (fiber-optic PWM and IDC), the fiber-optic wavelength shift temperature sensor, the fiber-optic Sagnac loop sensor, and the probe type fiber-optic temperature sensor were 5–100 °C, 5–100 °C, 26–40 °C, 20–50 °C, and 42–90 °C. Therefore, the sensitivities and dynamic ranges of the proposed temperature sensors were better than those of the other above-mentioned temperature sensors. In addition, the linearity i.e. the correlation coefficient R^2^ of the proposed fiber-optic PWM as well as IDC temperature sensing system were higher than the fiber-optic wavelength shift temperature sensor [38], fiber-optic Sagnac loop sensor [39], and probe type fiber-optic temperature sensor.

## 5. Conclusions

In this study, we proposed highly sensitive, wide dynamic range temperature sensors that are based on fiber-optic PWM and the capacitance variation principle. A well-known thermochromic dye (i.e., Rhodamine B) was incorporated into PVC and a DMAC solution to create the temperature-sensitive sensing membrane, which was then deposited on the side-polished single mode optical fiber device and in the IDE to prepare fiber-optic and IDC temperature-sensing elements, respectively. The pulse width of the received sensing signal and the IDC capacitance change with change in temperature. As a result, the amplitude of the received sensing signal changes. The proposed sensors offer a wide dynamic range with linear sensing performance. The correlation coefficient (R^2^) values were ~0.992. The sensors have highly stable sensing capabilities, high reproducibility, low fabrication costs, and real-time sensing responses. Moreover, the cost of the electronic components used to design the electronic circuitry is low and they are available at local electronic suppliers. In future studies, we will use temperature-sensing membranes based on different thermochromic compounds to increase the number of sensing elements in the array. We also plan to design a fiber-optic distributed temperature sensor.
